# Interactions between photodegradation components

**DOI:** 10.1186/1752-153X-6-100

**Published:** 2012-09-11

**Authors:** Yadollah Abdollahi, Azmi Zakaria, Khamirul Amin Matori, Kamyar Shameli, Hossein Jahangirian, Majid Rezayi, Tahereh Abdollahi

**Affiliations:** 1 Material Synthesis and Characterization Laboratory, Institute of Advanced Technology, Universiti Putra Malaysia, 43400 UPM Serdang Selangor, Malaysia; 2 Department of Chemistry, Faculty of Science, Universiti Putra Malaysia, 43400 UPM Serdang Selangor, Malaysia

**Keywords:** Cross-product effects, Modeling, Multivariate, Photocatalyst, Photodegradation, Variable-interaction, ZnO

## Abstract

**Background:**

The interactions of *p*-cresol photocatalytic degradation components were studied by response surface methodology. The study was designed by central composite design using the irradiation time, pH, the amount of photocatalyst and the *p*-cresol concentration as variables. The design was performed to obtain photodegradation % as actual responses. The actual responses were fitted with linear, two factor interactions, cubic and quadratic model to select an appropriate model. The selected model was validated by analysis of variance which provided evidences such as high F-value (845.09), very low P-value (<.0.0001), non-significant lack of fit, the coefficient of R-squared (R^2^ = 0.999), adjusted R-squared (R_adj_^2^ = 0.998), predicted R-squared (R_pred_^2^ = 0.994) and the adequate precision (95.94).

**Results:**

From the validated model demonstrated that the component had interaction with irradiation time under 180 min of the time while the interaction with pH was above pH 9. Moreover, photocatalyst and *p*-cresol had interaction at minimal amount of photocatalyst (< 0.8 g/L) and 100 mg/L *p*-cresol.

**Conclusion:**

These variables are interdependent and should be simultaneously considered during the photodegradation process, which is one of the advantages of the response surface methodology over the traditional laboratory method.

## Background

Advanced oxidation processes (AOPs) are physicochemical procedures, which designed to remove environmental organic and inorganic pollution. Photocatalysis, the current interest of AOPs, is applied for decontamination the pollutions [1-4]. The photocatalysis, under suitable light illumination, produces hydroxyl radical (^●^OH) and hole (h^+^) which are powerful and non-selective oxidants to degrade a variety of organic compounds [5-7]. Since the photocatalytic degradation (photodegradation) is dependent on several parameters including irradiation time, pH, photocatalyst and pollution concentration, it need to study the relationship between the variables during the process [8,9]. In the design of experiments, the independent variables are controlled to determine the relationship to an observable phenomenon [10]. The single variable (one-variable-at-a-time) method considers the photodegradation process as a projection while the multivariate method generalizes the observation of the photodegradation [11]. Therefore, the multivariate, which, increases the dimension of the system and produces more generalized results is preferred in comparing with the single variable approach. Recently the semi-empirical methods were used as an efficient technique to apply multivariate modeling for the photodegradation by response surface methodology (RSM) [12-18], however, no study has yet been conducted on the parameters interaction. This work looks at the parameters interaction of *p*-cresol photodegradation as a sample of organic pollution in present of ZnO as a photocatalyst by the RSM. The interaction between irradiation time, pH, photocatalyst loading, and *p*-cresol concentration (as variables) were investigated during the photodegradation process.

## Experiment

### Empirical methodology

To study of the interactions or cross-product effects between the photodegradation parameters, the experiments were designed with multi factors (Table 1) by the RSM. The designed experiments were performed according to previous work procedure [19] to obtain actual responses that used as input for Design-Expert 8 software. To detect and suggest a valid model, the actual responses were fitted with existing linear, two factor interactions (2FI), cubic and quadratic model by central composite design (CCD). Based on suggested model, the quadratic model was selected to continue the progress. The selected model was validated by a few numbers of statistical evidences in the analysis of variance (ANOVA). The evidences were including Fisher variation ratio (F-value), probability value (P-value), Lack of Fit, coefficient of determination R-squared (R_d_^2^), adjusted R-squared (R_Adj_^2^), predicted R-squared (R_Pred_^2^) and adequate precision (PRESS). PRESS is a signal-to-noise ratio, which compares the range of the predicted values at the design points to the average prediction error. The ratios greater than 4 indicate adequate model discrimination [20]. R_Adj_^2^ and the R_Pred_^2^ are measurements of the amount of variation around the mean and new explained data respectively. F-value is a statistically valid measure of how well the factors describe the variation in the data about its mean while P-value represents the degree of significance of each variable. Most of these parameters are clearly defined in experimental design texts [20]. The validated model is able to predict the interactions between variables such as X_1_X_2_ (Table 1) during the photodegradation process.

**Table 1:** Independent variables and their levels employed in the central composite design

	** Variables**	**Units**	**Level of Variables**	
			**Low**	**High**
X_1_	Irradiation time	min	0	360
X_2_	*p*-cresol	Mg/L	0	75
X_3_	Photocatalyst	g/L	0.5	4
X_4_	pH	-	4	10

## Analysis of the results

### The model validation

**Figure 1:**
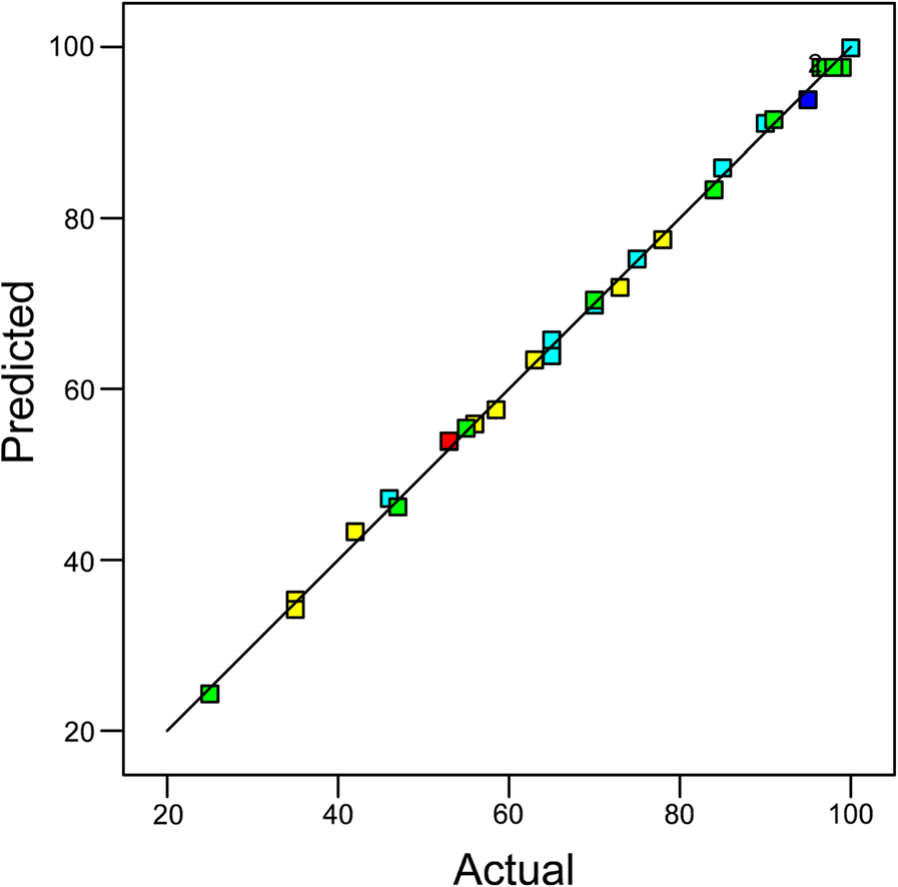
**The scatter plot of predicted values versus actual values of *p*-cresol photodegradation.** The coefficient of R-squared (R^2^) is 0.9987.

The selected quadratic model displayed expresses the relationship between responses of actual variables and the variables themselves (Eq. 1).

(1) $$\begin{array}{ll} \;Y= & -602.66146+1.004X_{1}+108.590X_{2}\\ & +113.696X_{3}+1.478X_{4}-1.736\times 10^{-3}X_{1}X_{2}\\ & +0.072X_{1}X_{3}+6.458\times 10^{-4}X_{1}X_{4}-4.375X_{2}X_{3}\\ & -2.50\times 10^{-3}X_{2}X_{4}-0.393X_{3}X_{4}-1.996X_{1}^{2}\\ & -6.416X_{2}^{2}-20.746X_{3}^{2}-9.498X_{4}^{2} \end{array}$$

where ‘Y’ is photodegradation % and the actual values of the variables X_1_, X_2_, X_3_ and X_4_ are shown in Table 1. As observed, the ANOVA of the model indicated that high model F-value, the values of Prob.>.F, the Lack of Fit, the determination coefficient, the R_Adj_^2^, the R_Pred_^2^ and the adequate precision were 845.09, 0.0001 (<.0.0500), not significant, R^2^.=.0.999, 0.998, 0.994 and 95.94, respectively. Moreover, Figure 1 shows the actual values versus predicted values of the photodegradation, which indicates an excellent agreement between actual and predicted values. As observed, the validity (significance and adequacy) of the model was confirmed by the reasonable evidence.

### Interaction of variables

**Figure 2:**
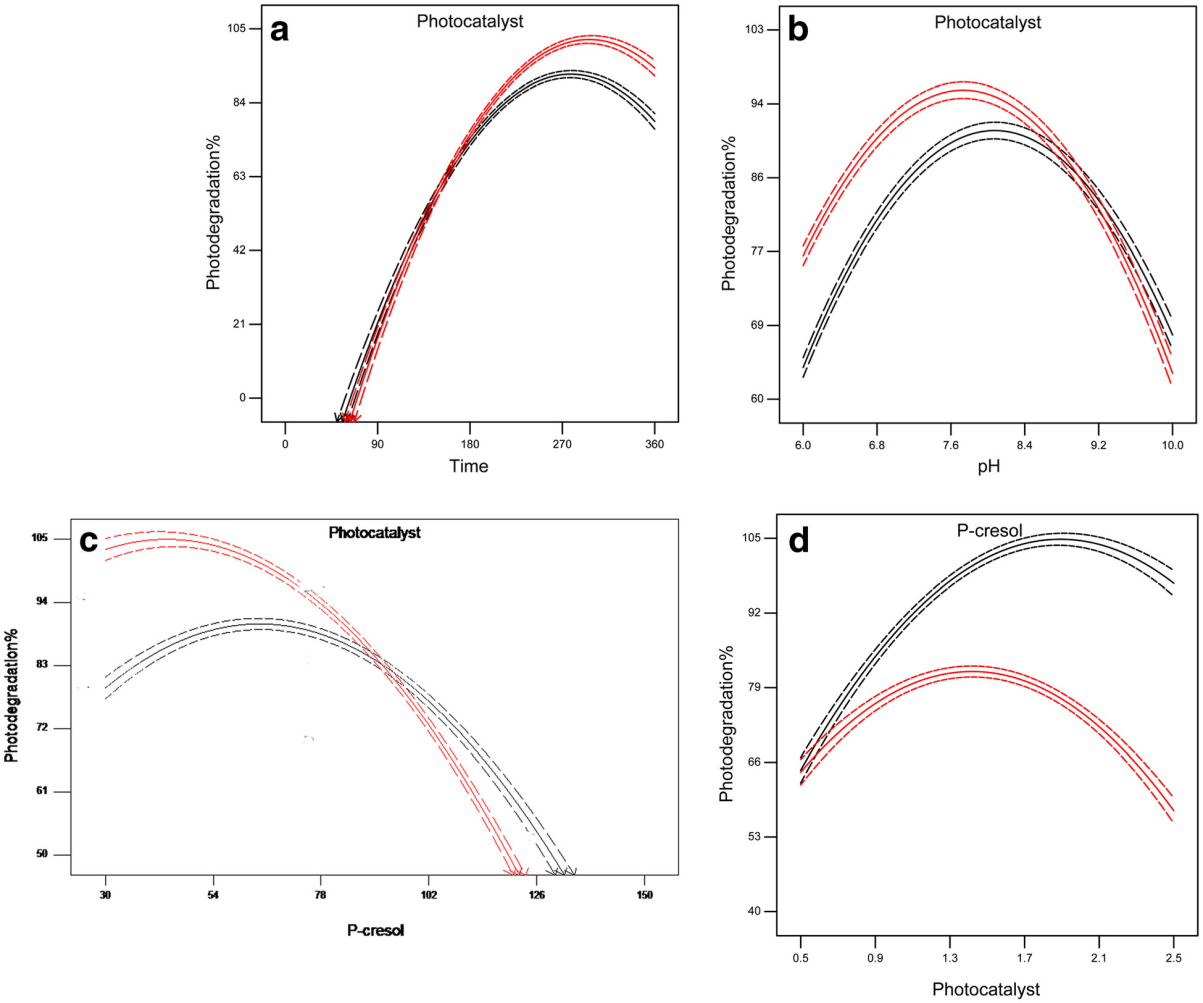
**Interaction between two parameters of p-cresol photodegradation while other two variables kept constant during the process, (a) interaction between irradiation time and photocatalyst amount, (b) interaction between pH and photocatalyst, (c) interaction between concentration of p-cresol and photocatalyst.** The red and black lines on the Figures 2 and 3 are the above axes and below axes as showed on the figures respectively which provided by the software (RSM).

**Figure 3:**
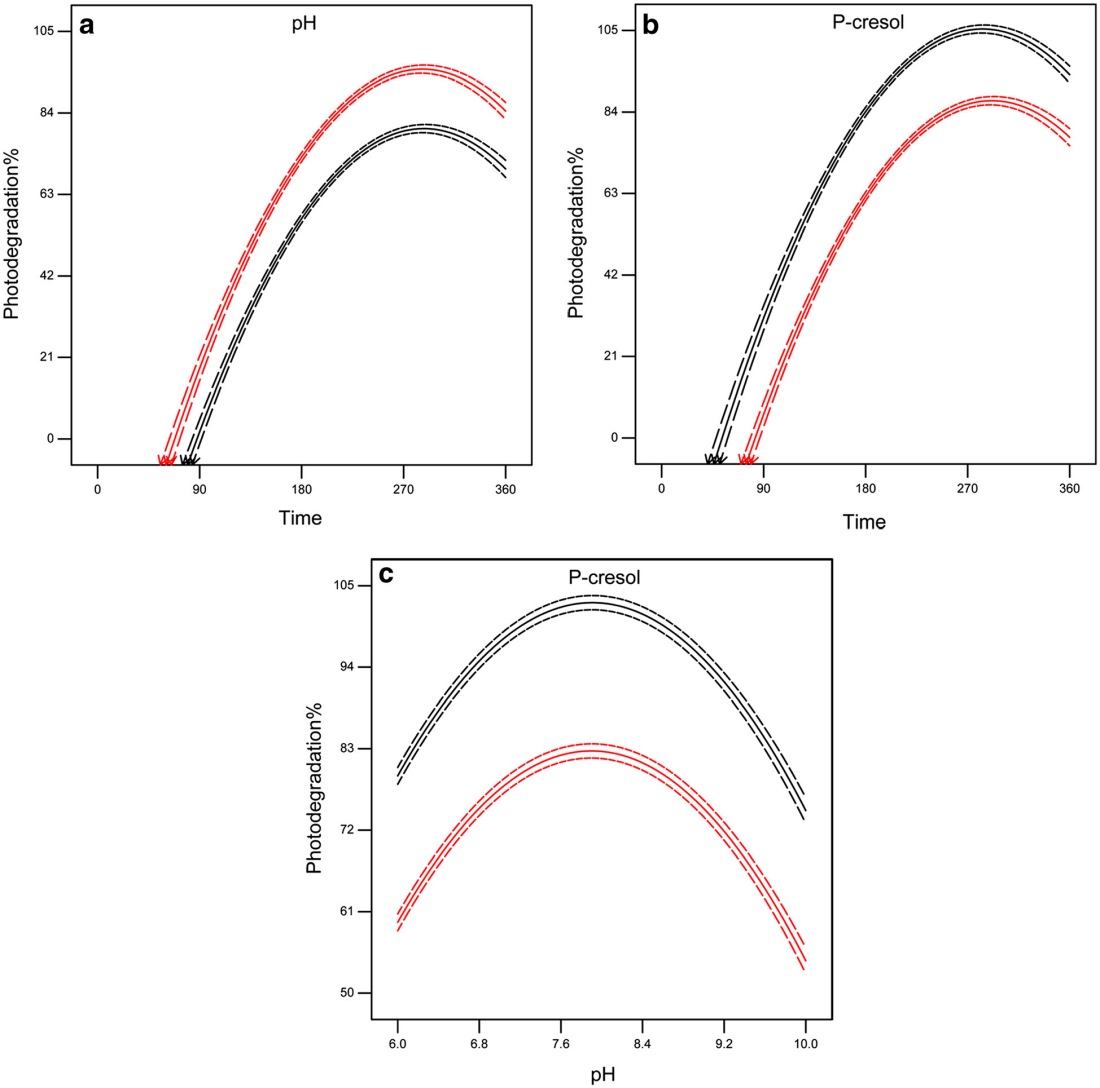
**The simultaneous behavior of the variables during *p*-cresol photodegradation in the quadratic model, (a) behavior irradiation time and pH, (b) behavior concentration of p-cresol and irradiation time, (c) behavior of pH and concentration of p-cresol.** The red and black lines on the Figures 2 and 3 are the above and below axes as showed on the figures respectively which provided by the software (RSM).

The validated model (Eq. 1) shows interaction effects of variables on the photodegradation % (Y). The interaction parameters are demonstrated by X_1_X_2_, X_1_X_3_, X_1_X_4_, X_2_X_3_, X_2_X_4_, X_3_X_4_ which, presented in Table 1. The parameters were related by coefficients and the signs (+, −) in the model. The coefficients indicate the specific weight of the parameters in the model. While the signs (+) and (−) affect the synergistic and antagonistic effects of variables on the response (Y). The coefficients indicated the weight of variables in the model, which determine the importance roles of the parameters in the photodegradation. As the coefficients illustrated, the importance of the interactions are X_1_X_2_.>.X_2_X_3_.>.X_3_X_4_.>.X_1_X_3_.>.X_2_X_4_.>. X_2_X_4_. The synergistic effect in the model translates to an improvement the photodegradation while the antagonistic effects were recessive. As observed, the interactions X_1_X_3_ and X_1_X_4_ had synergistic effect while X_1_X_2_, X_2_X_3_, X_2_X_4_ and X_3_X_4_ were antagonistic effect. Moreover, the model is capable to present the interactions graphically during the photodegradation process. In each case, the graphs display behavior of the two parameters while other two variables kept constant in the process. It should be mentioned that the red and black lines on the Figures 2 and 3 are the above and below axes as showed on the figures respectively which provided by the software (RSM). Figure 2a shows the interaction between irradiation time and amount of photocatalyst while pH and concentration of cresol was 7.5 and 75 mg/L respectively. As observed, the variables are interdependent below 180 min of irradiation. Therefore, these variables are not independence over the study time (0–180 min). To consider the interactions, it is necessary to study simultaneously several variables (multi variation) during the photodegradation, which is one of the advantages of the response surface method over the traditional laboratory method. The interaction between pH (6 – 9) and photocatalyst (0.5 – 2.5 g/L) was simultaneously studied with constant *p*-cresol concentration (75 mg/L) at the end of irradiation time (Figure 2b). The variables were dependent above 2.0 to 2.5 g/L of photocatalyst and pH 9 to 10. This can be attributed to the shift in surface characteristics above pH 9 [21], which also mean interdependence of these variables dependent in the range of pH. However, the variables were independent within 0.5 g/L to 2.0 g/L photocatalyst concentration and pH 6 to 9 which, may be due to charge of photocatalyst, which is positive under zero point charge [21]. Moreover, Figure 2c represents the simultaneous behavior of *p*-cresol concentration (0 – 75 mg/L) and photocatalyst amount (0.5 – 2.5 g/L) in constant pH (7.5) and at the end of irradiation time (240 min). As illustrated, the interaction was observed at 100 mg/L of *p*-cresol concentration. On the other view, the variables had interaction at minimal amount of photocatalyst (< 0.8 g/L) and concentration of *p*-cresol (Figure 2d). It may be related to the probability interaction between *p*-cresols and photocatalyst surface [22,23].

Figure 3 shows, the simultaneous behavior of *p*-cresol photodegradation variables during irradiation time. It may be observed from Figures 3a, b and c, that there are no clear interactions between irradiation time with pH, irradiation time with *p*-cresol and pH with *p*-cresol. Therefore, these variables can be independently investigated.

## Conclusion

The study of four photodegradation variable’s behavior including irradiation time, pH, amount of photocatalyst and *p*-cresol concentration, experiments were designed by central composite design (CCD). The design was performed to obtain actual responses. The actual responses were fitted with linear, two factor interactions (2FI), cubic and quadratic model by RSM to obtain an appropriate model. The model was validated by analysis of variance (ANOVA). The obtained visual results from the validated model demonstrated that there is no clear interaction between irradiation time with pH, *p*-cresol with irradiation time, and pH with *p*-cresol. Therefore, these variables can be independently investigated. However, the component of photocatalyst amount interacted with other variables as following. The component had interaction with irradiation time under 180 min of the time while the interaction with pH was above pH 9. Moreover, photocatalyst and *p*-cresol had interaction at minimal amount of photocatalyst (< 0.8 g/L) and 100 mg/L concentration of *p*-cresol. Therefore, these variables should be simultaneously considered during the photodegradation process.

## Competing interests

Are there any non-financial competing interests (political, personal, religious, ideological, academic, intellectual, commercial or any other) to declare in relation to this manuscript? The author(s) declare that they have no competing interests.

## Authors’ contributions

Yadollah Abdollahi (AB, JY, MT) Azmi Zakaria (FG) Khamirul Amin Matori (FG) Hossein Jahangirian (JY) Kamyar Shameli (JY) Majid Rezayi (JY) Tahereh Abdollahi (JY, MT) AB carried out the catalyst design and ligand screening studies. JY carried out the synthesis, purification and characterization of the compounds. MT carried out the computational experiments. FG conceived of the study, and participated in its design and coordination and helped to draft the manuscript. All authors read and approved the final manuscript.
